# Assessment of Right Ventricular Function—a State of the Art

**DOI:** 10.1007/s11897-023-00600-6

**Published:** 2023-06-05

**Authors:** Abdul Hameed, Robin Condliffe, Andrew J. Swift, Samer Alabed, David G. Kiely, Athanasios Charalampopoulos

**Affiliations:** 1 Sheffield Pulmonary Vascular Disease Unit, Royal Hallamshire Hospital, Sheffield, UK; 2 Department of Infection, Immunity and Cardiovascular Disease, University of Sheffield, Sheffield, UK; 3 INSIGNEO, Institute for in silico Medicine, University of Sheffield, Sheffield, UK; 4 NIHR Sheffield Biomedical Research Centre, Sheffield, UK

**Keywords:** Right ventricular function, Pulmonary hypertension, Right ventricular ejection fraction, Right ventricular-pulmonary arterial coupling, Right heart catheterisation

## Abstract

**Purpose of Review:**

The right ventricle (RV) has a complex geometry and physiology which is distinct from the left. RV dysfunction and failure can be the aftermath of volume- and/or pressure-loading conditions, as well as myocardial and pericardial diseases.

**Recent Findings:**

Echocardiography, magnetic resonance imaging and right heart catheterisation can assess RV function by using several qualitative and quantitative parameters. In pulmonary hypertension (PH) in particular, RV function can be impaired and is related to survival.

**Summary:**

An accurate assessment of RV function is crucial for the early diagnosis and management of these patients. This review focuses on the different modalities and indices used for the evaluation of RV function with an emphasis on PH.

## Introduction

### Anatomy of the Right Ventricle

The right ventricle (RV) has a very distinct anatomy compared to the left (LV). It consists of 3 segments as follows: the inlet which includes the tricuspid valve (TV), the outlet or infundibulum with the pulmonary valve and a trabeculated apex [1]. The RV has at least 3 papillary muscles, a moderator band which incorporates the right bundle branch [2] and a myocardial fold called *crista supraventricularis*. RV myocardial fibres are arranged circumferentially in the superficial and longitudinally in the deep layers leading to a contraction from inlet to outlet and from the free wall to the septum. RV contraction relies more on longitudinal shortening than twisting and rotational movements as in the LV [3]. Normal coronary perfusion to the RV occurs during both systole and diastole in contrast with the LV which is perfused mainly in diastole. In utero RV and LV wall thickness are equal, however, once the umbilical cord is clamped at birth pulmonary vascular resistance (PVR) reduces rapidly and RV wall thickness reverses [4, 5]. Hence, the RV is a thin-walled and crescent-shaped ventricle compared to a thick-walled and bullet-shaped LV [6]. This feature makes the RV respond better to volume overload but worse to pressure loading, unlike the LV. Both ventricles share the septum (IVS) and are contained within the same pericardium; these commonalities determine their interdependence [7].

### Physiology

RV function should be examined along with pulmonary arterial (PA) function [8•]. RV and PA form a cardiopulmonary unit the function of which is characterised by two components: RV contractility and PA load, which is also known as RV afterload. In a normal-functioning RV, these two components are ‘coupled’. This ‘RV-PA coupling’ secures an efficient energy transfer of RV load to arterial load. The gold-standard metric to express RV contractility is end-systolic elastance (Ees). Elastance describes the change in pressure for a given change in volume and is a property of elastic chambers. Ees is load-independent and is determined by the contractile force of the myocyte and cardiac muscle hypertrophy [9].

PA load consists of two elements as follows: a steady and a pulsatile load. PVR is an expression of the steady load and pulmonary artery compliance (PAC) of the pulsatile load. PVR and PAC are inversely related [10]. PAC is a measure of PA distensibility and may be more sensitive to early disease [11, 12]. The calculation of a total PA load incorporating all components is challenging but is best represented by effective arterial elastance (Ea). There are different ways to calculate Ees, PVR, PAC, Ea and RV-PA coupling, which are described under ‘Invasive Haemodynamics’ in this review.

### Pathophysiological Changes in a Pressure-Loaded RV

This review will focus on the assessment of RV function in pulmonary arterial hypertension (PAH) which is the archetypical example of a pressure-loaded RV. In PAH, the proliferative changes in the small pulmonary arteries may lead to a decrease in PAC and a subsequent increase in PVR. The RV adapts to the increased afterload by concentric hypertrophy which can raise its contractility 4 to 5-fold, and thus ‘RV-PA coupling’ will be maintained [13]. However, as PAH advances and further RV hypertrophy is not feasible, the RV starts dilating as this is the only way to maintain stroke volume (SV) via the Frank-Starling mechanism [14•]. RV dilatation increases wall stress and oxygen consumption and triggers ischaemia via two mechanisms as follows: elevated oxygen demands and reduced perfusion in the context of increased intramural pressure [15, 16]. Another consequence of RV dilatation is the development of functional tricuspid regurgitation (TR) via TV annulus stretching, which causes RV volume loading and further dilatation. In addition, IVS shifts towards the LV in systole leading to interventricular dyssynchrony, LV underfilling and myocardial cell atrophy [17]. Thus, in PH it is more accurate to refer to biventricular rather than solely RV dysfunction and failure.

### Aetiology of Right Heart Failure

Right heart failure (HF) is a syndrome characterised by alteration in structure and/or function of the RV-PA unit leading to suboptimal blood flow to the pulmonary circulation [18, 19]. Its causes are summarised in Table 1. It is not uncommon for more than one pathology or mechanisms to be present.

**Table 1:** Causes of right heart failure

**Aetiology of right heart failure**	
1. **Pressure overload**	
i) Acute: PE ii) Chronic: PH, PA or PV stenosis [20]	
2. **Volume overload**	
i) TR: *structural* (e.g. Ebstein’s anomaly [21], carcinoid, endocarditis, and flail valve) and *functional* (e.g. PH and chronic AF) ii) PR: *structural* (e.g. carcinoid and endocarditis) and *functional* (e.g. PA dilatation) iii) Left-right shunt (ASD and PAPVD) iv) High output status (e.g. anaemia, thyrotoxicosis, liver cirrhosis, and Paget’s)	
3. **Myocardial disease**	
i) RV myocardial infarction [22] ii) Arrhythmogenic RV cardiomyopathy [23] iii) Myocarditis [24] iv) Dilated cardiomyopathy v) Takotsubo cardiomyopathy vi) Post-surgery [25, 26] vii) Uhl’s anomaly (aplasia or hypoplasia of RV myocardium) [27] viii) Endomyocardial fibrosis [28] ix) Amyloidosis [29] x) Chagas disease	
4. **Pericardial disease**	
i) Constriction ii) Tamponade	
5. **Iatrogenic (via volume or pressure overload)**	
Excessive volume loading, mechanical ventilation	
6. **Mixed (e.g. pressure and volume overload)**	

*PE* pulmonary embolism, *PH* pulmonary hypertension, *PA* pulmonary artery, *PV* pulmonary valve, *TR* tricuspid regurgitation, *AF* atrial fibrillation, *PR* pulmonary regurgitation, *ASD* atrial septal defect, *PAPVD* partial anomalous pulmonary vein drainage, *RV* right ventricular

## Assessment of RV Function

### Echocardiography

Echocardiography is an established tool for the evaluation of cardiac structure and function and can be used for diagnosis, monitoring and treatment guidance. Its widespread availability, versatility and relative ease of use makes it a fundamental investigation. Its limitations include incomplete visualisation of all aspects of the RV, poor echogenicity in some subjects and a lower repeatability than cardiac MRI. In addition, loading conditions (e.g. high cardiac output status and significant TR), ventilatory failure and the use of inotropes or invasive ventilation may influence RV function and should be considered during its evaluation. Echocardiography can provide qualitative and quantitative assessment of RV function. 2D imaging, M-mode, Doppler, Tissue Doppler Imaging (TDI) and colour flow mapping are standard approaches, whilst strain and 3D echocardiography have garnered a significant evidence base for diagnosis and prognostication. Often no single parameter is sufficient, and a holistic approach [20] is required, whilst protocols and datasets for RV assessment have been published [21–24].

The complex geometry of the RV and its retrosternal position make its imaging challenging. In standard 2D imaging, the RV focused apical 4-chamber view (RVf4C) rather than the conventional 4-chamber view should be used as the standard tomographic plane to measure linear metrics of RV size and function [21, 24–26]. In this view, RV size is systematically larger with lower variability, compared to the conventional view [26]. A basal RV diameter > 42 mm and/or RV:LV ratio > 1 indicate RV dilatation. More recent data support a normal basal RV size > 47 mm in males and 43 mm in females [21•]. Metrics of RV function are discussed below. Although not discussed in this review, continuous wave (CW) Doppler of tricuspid and pulmonary regurgitant velocities allow estimation of PA pressures and form an essential part of RV assessment. Furthermore, pulse wave (PW) Doppler of the RVOT can allow detection of a raised PVR; short PA acceleration time and mid-systolic notching are indicative of increased wave reflection [27,22•, ]. In a comprehensive RV assessment, it is also important for the compartments proximal (right atrium (RA)) and distal (PA) to the RV to be evaluated, as well as the left heart and pericardium.

### Assessment of Regional RV Function

#### TAPSE

**Fig. 1:**
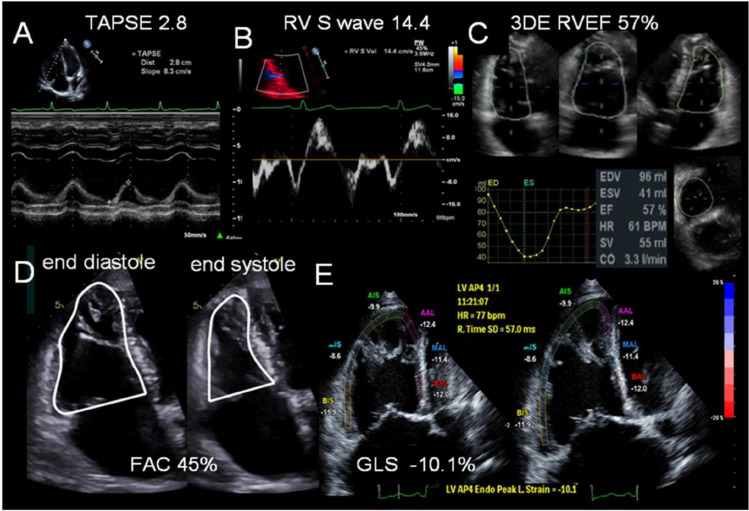
Echocardiographic parameters for the quantitative assessment of RV function. **A** Tricuspid annular plane systolic excursion (TAPSE), **B** right ventricular systolic wave velocity measured with Tissue Doppler Imaging (RV S′), **C** right ventricular ejection fraction measured by 3D-echocardiography (3DE RVEF), **D** fractional area change (FAC), **E** 2D right ventricular global longitudinal strain (GLS). *Original image republished without need for permission under a Creative Commons Attribution-Non-Commercial-No Derivatives 4.0 International License from Rana et al.* [37]

Tricuspid annular systolic plane excursion (TAPSE) is defined as the displacement (distance usually expressed in mm) of the lateral TV annulus during systole with M-mode echocardiography (Fig. 1). It is simple and reproducible but is load- and angle-dependent. It is measured from edge to edge with a high sweep speed (100 mm/s). In the setting of TR, it can become pseudo-normalised due to volume loading, and other indices such as strain [28, 29] or RV ejection fraction (RVEF) [30, 31] are recommended. An abnormal TAPSE is defined as < 17 mm. In our experience, TAPSE is often the last metric of RV function to worsen in PH, despite a severe reduction in radial function which usually precedes. TAPSE has been shown to correlate closely with RVEF determined by radionucleotide ventriculography [32] and MRI [33], and it is a strong prognostic marker in PH and other cardiovascular diseases [34–36]. However, it can only assess the longitudinal function of the basal RV free wall and not global RV function.

#### RV Systolic Wave Velocity (S′)

S′ is the peak systolic velocity of the lateral TV annulus (Fig. 1). It is measured with PW-TDI. It is angle- and load-dependent like TAPSE, and the ultrasound beam should align with the lateral TV annulus. It is a measure of the longitudinal function of the RV base. It has been shown to moderately correlate with MRI-derived RVEF [33] and has prognostic value. An abnormal S′ is defined as < 9.5 cm/s.

### Assessment of Global RV Function

#### RV FAC

RV FAC is evaluated in the RVf4C view. It requires manual tracings of RV endocardial area at end-systole and end-diastole (Fig. 1). It should include the papillary muscles, trabeculations and moderator band. FAC is defined as $\frac{\left( end- diastolic\ area\right)-\left( end- systolic\ area\right)}{end- diastolic\ area}$ and is expressed as a percentage. An abnormal FAC is defined as < 35% [24•], whereas British guidelines stipulate abnormal as < 30% in males and < 35% in females [21•].

#### RVLS

Strain is a dimensionless measure of myocardial deformation. RV longitudinal strain (RVLS) is the longitudinal shortening of fibres between the base and apex of the RV in systole and is expressed as a negative percentage [38]. It is measured by using 2D speckle tracking. It depends heavily on the imaging quality of the endocardial/myocardial borders and should be measured in the RVf4C view with a high frame rate. A standardised protocol for acquisition has been published [21, 25, 39]. Global RVLS can be measured by using either three segments (RV free wall) or six segments (free wall and IVS), as these are more standardised than segmental values. RVLS is less angle- and load-dependent, less affected by translational motion and more reproducible than TAPSE and S′, whilst it can identify subclinical dysfunction and has established prognostic utility [20, 25]. However, it neglects the contribution of the RVOT, and values are affected by vendor specific software. At present, there is no universally agreed reference range [25, 40–43]; however, RVFWLS > −20% can be regarded as abnormal with values > −15% identifying those with severe reduction [25•]. RVLS is the best echocardiographic correlate of MRI-derived RVEF [44–46]. RVLS has demonstrated diagnostic and prognostic roles in heart failure [25, 47, 48], PH [49–56], congenital heart disease, cardiomyopathies and tricuspid regurgitation [57–60]. 3D RV strain imaging is conceptually appealing [61–63], but its clinical utility is less well established.

#### 3DE

Over the past decade improvements in 3D echocardiography (3DE) have led to its incorporation in the assessment of the left heart [64]. This has also led to a growing dataset establishing 3DE as a promising novel way of RV imaging [20, 65]. 3DE can circumvent most of the limitations of 2D parameters and calculate RVEF which is the best measure of global RV function [66–68]. 3DE has the unique advantages of simultaneously allowing assessment of several aspects of the right heart including volumes, function, ejection fraction and TV morphology. 3DE-derived RVEF closely correlates with MRI [69, 70]. In PH, 3DE has characterised RV geometry [70], patterns of RV remodelling [63], haemodynamics [71] and RV-PA coupling [72] and has been correlated with outcomes [73–75]. Limitations of 3DE include its dependency on good image quality (up to 25% cases are not feasible), regular heart rates, need for specific expensive hardware and software, time and expertise [20, 65, 76].

#### Other indices

The myocardial performance index (MPI) has generally fallen out of favour for TAPSE, S′ and FAC but is still used in some institutions. It should be measured with PW-TDI on the lateral TV annulus. It is defined as the ratio of isovolumic time (contraction and relaxation) to ejection time and an abnormal value is > 0.55. It is load-dependent and invalid in high RA pressures [21, 23]. Assessment of diastolic RV function is less well validated but includes inferior vena cava (IVC) profile, RA size and function (area and strain), PW Doppler of RV inflow, hepatic vein flow and PW-TDI of the TV annulus [21, 24].

### Echocardiographic Assessment of RV-PA Coupling

#### TAPSE:PASP Ratio

This ratio was first proposed and evaluated as a surrogate of RV-PA coupling in a series of patients with HF and showed a prognostic role independent of the severity of LV dysfunction [77–79]. ^.^TAPSE:PASP ratio has also been tested in pre-capillary PH where it correlated with haemodynamics and was an independent predictor of survival [80, 81]. TAPSE:PASP has been validated with invasive RV pressure-volume (P-V) analysis in a small group of patients with severe PAH [82]. Consequently, TAPSE:PASP ratio has been incorporated into the risk assessment algorithm of the 2022 ESC/ERS PH Guidelines [83•] and has a potential role in suspected PH in patients with systemic sclerosis [84]. Its limitations relate to incomplete/inaccurate estimation of PASP from TR jet velocity and IVC profile.

#### RVFWLS:PASP Ratio

A recent study has highlighted an independent prognostic role for the baseline RV free wall longitudinal strain RVFWLS:PASP ratio in treatment-naive patients with precapillary PH [85]. A preliminary analysis by an independent group has offered some retrospective validation for this parameter in a small cohort using invasive P-V loop analysis [86].

### Stress Echocardiography

All of the metrics described above can be evaluated at rest and after peak stress (exercise or with dobutamine). Most of the data so far have been derived from healthy volunteers, but there are several small to medium-sized studies which have evaluated the prognostic role of RV contractile reserve in patients with PH, valve disease and HF [87].

### MRI

#### Cine Cardiac MRI

**Fig. 2:**
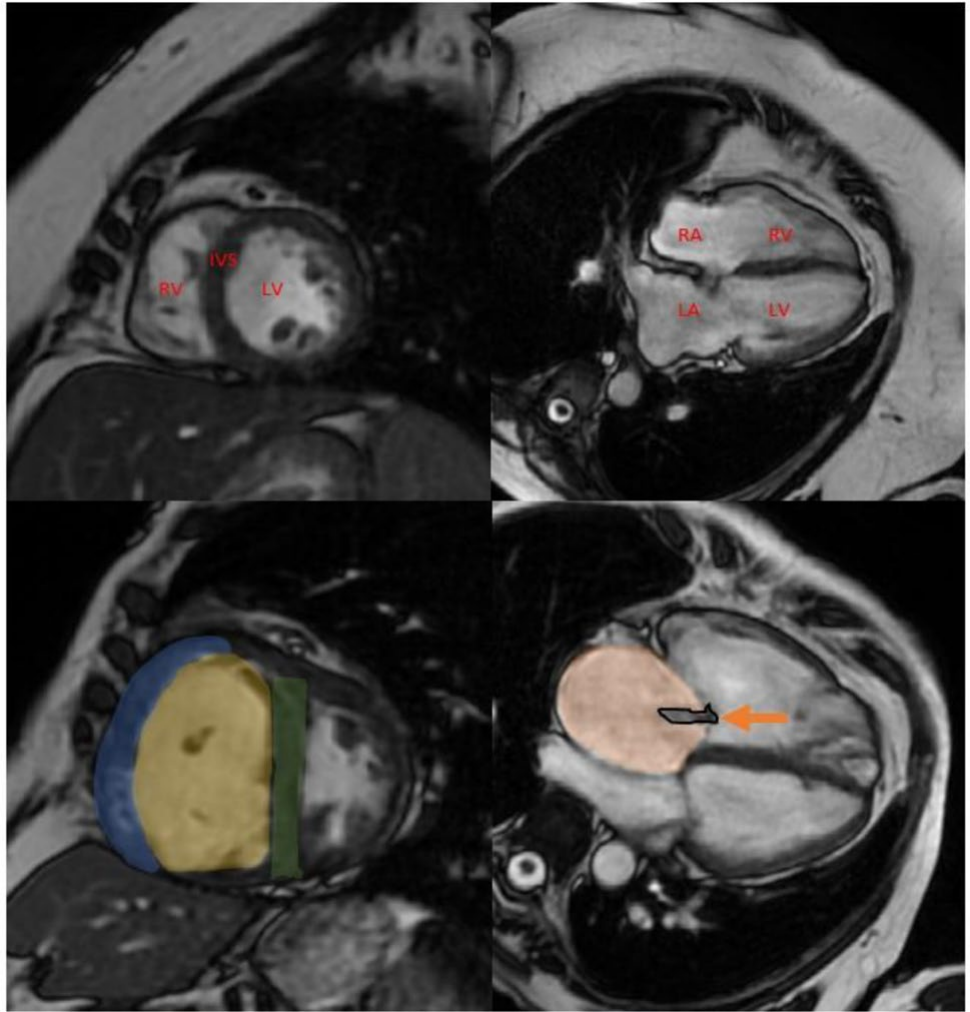
Cardiac MRI cine images (short-axis: left, four chamber: right). *Top row:* normal MRI. RV; right ventricle, LV; left ventricle, IVS; interventricular septum, RA; right atrium, LA; left atrium. *Bottom row:* pulmonary arterial hypertension features including a hypertrophied RV myocardium (blue), dilated RV chamber (yellow), IVS straightening (green), RA enlargement (orange) and tricuspid regurgitation jet (arrow)

In PH, RV failure is the main determinant of death [83, 88]. MRI is considered the gold-standard for the assessment of RV size and function. The best index of global RV function is RVEF [89]. The volume of the cardiac chambers is built up using multiple slice positions in the short axis orientation with area measurements extrapolated to volume, mass and function measurements. The sequence used is balanced steady state free precession imaging [90]. RV volume and mass can be measured using the Simpson’s method, which involves tracing the RV endocardial border in multiple slices and calculating the total volume by summation. There are different cine views used in PH assessment, including but not limited to the 4-chamber view, short-axis view and axial views. In PH, cine MRI can detect RV dilatation and hypertrophy [91–94], IVS deviation towards the LV [95, 96] (Fig. 2) and PA dilatation and flow abnormalities [97–99]. Models combining these parameters have proven of value to diagnose PH at a tertiary referral setting [100, 101] and using modelling approaches [102]. A recent meta-analysis showed the prognostic power of MRI-derived RV parameters in PAH [103]. Adaptation of the RV to elevated afterload has been assessed by estimation of PA elastance and RV-PA coupling and predicts mortality [104–107]. Patients with RV dilatation without associated hypertrophy have a worse outcome than patients with hypertrophy [108, 109].

#### 4D Flow

Four-dimensional flow (4D) flow is an MRI technology that overcomes limitations of traditional 2D flow imaging in the evaluation of PH. Unlike conventional imaging, 4D flow provides a 3D visualisation of vascular flow and enables accurate assessment of vessel, transvalvular or intra-cavity flow, providing a more comprehensive description of complex flow changes in the pulmonary circulation. In the context of PH, 4D flow has been utilised to identify abnormal flow patterns in the main PA [110, 111], which are correlated with mean PA pressure (mPAP) [112] making its estimation possible. Furthermore, 4D flow allows the characterisation of other physiological vascular parameters, such as main PA wall shear stress, which can affect vascular remodelling and is typically reduced in PH [113]. Further studies are needed to determine the incremental role of 4D flow MRI assessment in PH management.

#### RV Strain Imaging

The quantification of myocardial deformation strain is an established MRI technique [114]. One method of strain analysis, called “feature tracking”, follows cardiac borders throughout the cardiac cycle on cine images [115, 116]. Biventricular strain impairment in PH can assist in the early detection of right and left heart dysfunction [117–119]. Feature tracking has been used to predict outcomes in patients with PH whilst reduced RV circumferential and longitudinal strain rates are associated with an impaired RVEF [120]. Reduced RA strain and phasic function are markers of disease severity, with decreased RA strain to be associated with decompensated RV function and stiffness [121, 122].

#### **LGE**

Late gadolinium enhancement (LGE) is an MRI technique that uses gadolinium’s paramagnetic properties to identify areas of myocardial fibrosis [123]. LGE is associated with poor outcomes and increased mortality in cardiomyopathies [124]. However, in PH LGE, particularly at the RV insertion points or IVS, appears to be a consequence of increased mechanical stress and RV remodelling rather than a sign of RV decompensation and poor prognosis [125, 126].

#### Myocardial T1 and ECV Mapping

Native myocardial T1 mapping is a pixel-by-pixel representation of the longitudinal relaxation times within a tissue, providing surrogate tissue characterisation data that are measured on a standardized scale [127–129]. T1 values post-gadolinium can be used to estimate extracellular volume (ECV), which is calculated by subtracting the T1 values of the myocardium and blood pool pre- and post-contrast, corrected for the haematocrit level [127]. Elevated T1 mapping values and ECV can indicate areas of oedema and fibrosis in the myocardium [140, 141]. Recent studies have investigated their clinical application in PH [142–147]. T1-times are elevated in PH, particularly at the RV insertion points, and are associated with an increased IVS angle and LV eccentricity [142, 145]. However, the diagnostic application of T1 mapping in PH remains limited [142, 144, 148].

#### Strengths, Limitations, and Future Perspectives

A major strength of MRI is its high repeatability with a great value in the assessment of cardiac changes in response to PAH therapy [149]. The 2022 ESC/ERS guidelines [93•] have included thresholds for key RV parameters for mortality prediction. MRI limitations include its high cost and reduced availability compared to echocardiography. The future of image acquisition and analysis is bright with the revolution in machine learning which has offered the potential to improve the speed of image acquisition [150], image quality [151] and automation of RV measurements and their conversion into text reports via natural language processing. Progress has also been made in PH with artificial intelligence (AI), which has demonstrated higher repeatability in measurements, closer correlations with invasive haemodynamics and better survival prediction [152, 153].

### Invasive Haemodynamics

Direct measurement of four pulmonary haemodynamic parameters — RA pressure (RAP), PAP, PA wedge pressure (PAWP) and cardiac output (CO) — via right heart catheterisation (RHC) allows assessment of RV preload, afterload, function and PAC (Table 2). RHC with a fluid-filled Swan-Ganz catheter is most commonly performed via the internal jugular vein but may also be performed via the brachial or femoral veins [154].

**Table 2:** Haemodynamic parameters derived from standard right heart catheterisation

Parameter	Definition
RAP/PAWP ratio	$\frac{\mathrm{RAP}}{\mathrm{PAWP}}$
Pulmonary vascular resistance (PVR)	$\frac{\mathrm{mPAP}-\mathrm{PAWP}}{\mathrm{CO}}$
Total pulmonary resistance (TPR)	$\frac{\mathrm{mPAP}}{\mathrm{CO}}$
Stroke volume (SV)	$\frac{\mathrm{CO}}{\mathrm{HR}}$
Stroke volume index (SVi)	$\frac{\mathrm{CI}}{\mathrm{HR}}$
PA compliance	$\frac{\mathrm{SV}}{\mathrm{PASP}-\mathrm{PADP}}$
RV stroke work index (RVSWi)	$\frac{(\mathrm{mPAP}-\mathrm{RAP})\;\times\;\mathrm{CI}\;\times\;0.0136}{\mathrm{HR}}$
PA pulsatility index (PAPi)	$\frac{\mathrm{PASP}-\mathrm{PADP}}{\mathrm{RAP}}$

*RAP* right atrial pressure, *PAWP* pulmonary arterial wedge pressure, *mPAP* mean pulmonary arterial pressure, *CO* cardiac output, *CI* cardiac index, *SV* stroke volume, *PASP* pulmonary arterial systolic pressure, *PADP* pulmonary arterial diastolic pressure, *PAP* pulmonary arterial pressure, *RV* right ventricular

#### RAP

RAP reflects central venous pressure but can also be increased in severe TR and impaired RV and RA compliance [155]. In the context of PAH, RV pressure and volume overload results in impaired filling leading to elevated RAP. In PAH, PAWP is higher than RAP until RV failure occurs at which point the RAP:PAWP will rise above 1. This ratio has been identified as a strong prognostic marker, outperforming RAP and several other haemodynamic parameters in two large cohorts of PAH patients [156].

#### CO

CO may be assessed via RHC by thermodilution (TD), the direct Fick (DF) or indirect Fick (IF) technique [157•]. TD involves injection of cooled saline into the proximal port of the Swan-Ganz catheter. The decrease in temperature between the RA and the distal thermistor produces a TD curve from which CO is computer-generated. DF and IF methods rely on the Fick equation:

$${\textrm{VO}}_2=\textrm{CO}\times \left(\textrm{Ca}\hbox{--} \textrm{Cv}\right)$$

where VO_2_ = oxygen consumption; *C*_a_ = arterial oxygen content = systemic oxygen saturation (SaO_2_, %) × haemoglobin (g/dL) × 1.34/100 and *C*_v_ = mixed venous oxygen content = mixed venous saturation (SvO_2_, %) × haemoglobin (g/dL) × 1.34/100. The DF method is recognised as the gold-standard method but relies on direct measurement of VO_2_ which requires specialised equipment in the catheter suite which is not widely available. The TD method has been shown to be a reliable method of measuring CO when compared with DF, even in patients with TR or low outputs [158, 159]. The IF method has poor accuracy and precision when compared to DF [159]. Current ESC/ERS guidelines recommend the use of either DF or TD techniques apart from patients with intra-cardiac shunts where TD is inaccurate [93•].

RV SV can simply be calculated by dividing CO by heart rate (*HR*). SV indexed for body surface area ($SVi=\frac{CI}{HR}$ ) has been found to outperform standard haemodynamics in large cohorts of PAH patients [160]. RV stroke work index (RVSWI) is a further derived parameter (*RVSWI* = 0.0136 × SVi × (*mPAP-RAP*)), which aims to reflect effective work performed by the RV in every cardiac cycle but has limited supportive data in PAH [161]. PA pulsatility index (PAPi) calculated by the equation ${PAP}_i=\frac{PASP- PADP}{RAP}$ , where *PADP* is *PA* diastolic pressure, relates the ability of the RV to produce SV to its filling pressure and has been shown to be strongly predictive of survival in patients with advanced HF, but its role in PAH is less clear [162].

#### RV Contractility

CO and derived haemodynamic parameters such as SVi and RVSWI as well as imaging-derived markers of RV systolic function such as TAPSE, FAC and RVEF are all load-dependent. In addition to preload and afterload, RV function is also determined by intrinsic contractility which relates to myocardial shortening and will be impacted positively or negatively by processes such as compensatory hypertrophy or diffuse fibrosis. Ees is the gold-standard measure for RV contractility but cannot be assessed via standard RHC [155, 163]. Instead, P-V loop assessment is performed which requires the use of a conductance catheter which measures both pressure and volume within the RV. Ees is calculated by performing multiple P-V loops at different preloads (achieved by progressive IVC compression with an intravenous balloon or Valsalva). The gradient of the line connecting the different end-systolic points (end systolic pressure-volume relationship) is Ees [164]. This multi-point technique is technically challenging and so a single-beat method has been developed [165]. Although the single-beat method is technically less challenging, it has poor reproducibility and prognostic capability in PH [166]. Instead of using a conductance catheter, Ees can also be obtained by combining RHC (to measure pressure) and CMR (to measure volumes) based on the equation: $Ees=\frac{mPAP}{ESV},$ where *ESV* is end-systolic volume [167].

#### RV Afterload

RV afterload consists of PVR and PAC. Total RV afterload can be described by *Ea* which can be derived from P-V loops: $Ea=\frac{end- systolic\ pressure}{SV}$ [165].

The relationship between RV load-independent function and RV afterload (RV-PA coupling) can therefore be described as the ratio between *Ees* and *Ea*. *Ees*:*Ea* should ideally be between 1 and 2. A recent study comparing RHC-derived *Ees*:*Ea* with CMR parameters demonstrated that *Ees*:*Ea* of < 0.8 was the best threshold indicating RV failure [168]. Interestingly a non-invasive CMR surrogate for RV-PA coupling, $\frac{SV}{ESV}$ was at least as useful in identifying impending RV failure [168].

PVR, which accounts for ≈75% of RV afterload, is calculated using the equation $PVR=\frac{mPAP- PAWP}{CO}$. This therefore relies on accurate measurement of both mPAP and PAWP. These should ideally be measured at end-expiration (functional residual capacity) when intra- and extra-thoracic pressures should be equivalent [157•]. Large respiratory swings in obesity, at exercise or in the presence of significant lung disease can make this difficult and in these situations, it is recommended that a computerised average is used [93•].

PAC may be calculated using the 2-element Windkessel model; however, the simplest way of estimating it is by using the equation $=\frac{SV}{PP}$ , where *PP* is pulse pressure i.e. PASP-PADP [169, 170]. Increased *PAC* leads to greater pulse wave reflection during systole which results in higher PASP requirements and hence higher RV energy requirements to create the same ejection.

The relationship between *PVR* and *PAC* (the resistance-compliance or RC time) represents the diastolic decay constant of PAP and in most conditions is constant, suggesting that most of both resistance and compliance occurs in the more distal vasculature [171].

## Conclusions

**Fig. 3:**
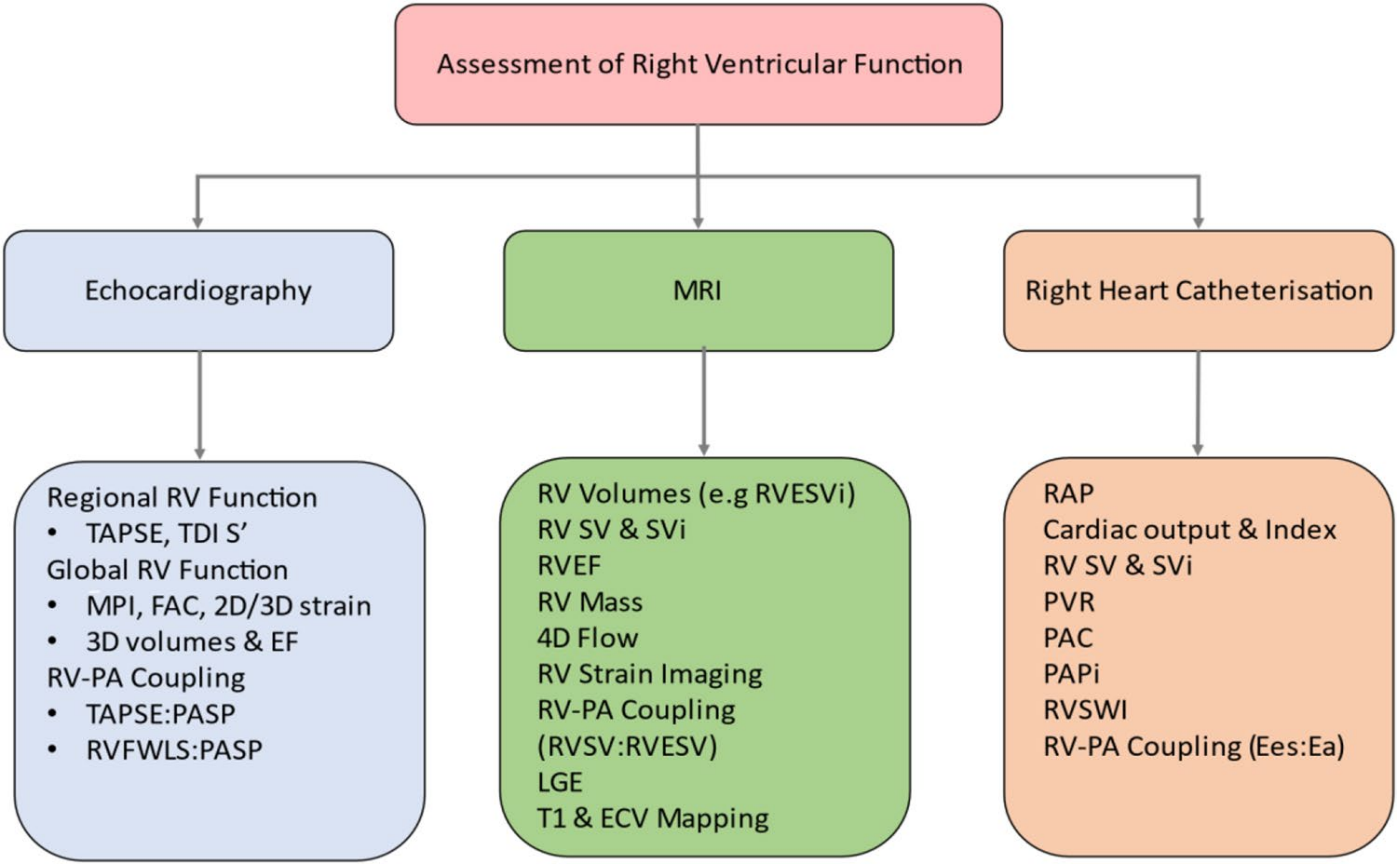
Modalities and parameters used for the assessment of right ventricular function. MRI, magnetic resonance imaging; RV, right ventricular; TAPSE, tricuspid annular plane systolic excursion; TDI S′, right ventricular systolic wave on Tissue Doppler Imaging; MPI, myocardial performance index; FAC, fractional area change; EF, ejection fraction; PA, pulmonary artery; PASP, pulmonary arterial systolic pressure; RVFWLS, right ventricular free wall longitudinal strain; RVESVi, right ventricular end-systolic volume index; SV, stroke volume; SVi, stroke volume index; RVSV, right ventricular stroke volume; RVESV, right ventricular end-systolic volume; LGE, late gadolinium enhancement; ECV, extracellular volume; RAP, right atrial pressure, PVR, pulmonary vascular resistance; PAC, pulmonary arterial compliance; PAPi, pulmonary arterial pulsatility index; RWSVI, right ventricular stroke work index; Ees, end-systolic elastance; Ea, arterial elastance

A systematic evaluation of RV size and function requires the use of several parameters derived from different invasive and non-invasive modalities. 2DE and 3DE as well as speckle tracking can provide a quantitative assessment of RV systolic performance. MRI-derived RVEF remains the gold-standard metric of RV global function and can be used in a serial manner to assess response to treatment and disease progression in PH. RHC is not only the gold-standard method for the diagnosis of PH, but it can also estimate CO, RV contractility and RV-PA coupling. It is imperative for the clinician to know the strengths and weaknesses of its test and use them in a complementary fashion accordingly (Fig. 3). Machine learning and AI methods may in the future help us to overcome the obstacles of RV complex shape and physiology and offer a more accurate and reproducible assessment of its global function.
